# Malignancies in systemic rheumatic diseases: A mini review

**DOI:** 10.3389/fimmu.2023.1095526

**Published:** 2023-02-28

**Authors:** Zhe Geng, Cong Ye, Xiaojian Zhu

**Affiliations:** ^1^ Department of Hematology, Central Hospital of Wuhan, Wuhan, China; ^2^ Department of Rheumatology and Immunology, Tongji Hospital, Tongji Medical College, Huazhong University of Science and Technology, Wuhan, China; ^3^ Department of Hematology, Tongji Hospital, Tongji Medical College, Huazhong University of Science and Technology, Wuhan, China

**Keywords:** malignancy, rheumatic disease, risk, disease-modifying anti-rheumatic drugs, mechanism

## Abstract

There is an increased risk of malignancies in patients with many systemic rheumatic diseases, which negatively impact on their quality of life. The risk and types of malignancies can differ by the type of rheumatic diseases. Possible mechanisms linking them are dynamic and complicated, including chronic inflammation and damage in rheumatic disease, inability to clear oncogenic infections, shared etiology and some anti-rheumatic therapies. Although certain disease-modifying anti-rheumatic drugs (DMARDs) have been proved to be potentially carcinogenic, the majority of them were not associated with increased risk of most malignancies in patients with systemic rheumatic diseases.

## 1 Introduction

It has been noted that the risk for the development of malignancies increases in patients with many systemic rheumatic diseases compared with general population (1). This may be related to the immunodeficiency and increased inflammatory burden caused by the pathobiology of the underlying rheumatic diseases, as well as other factors, such as environmental exposure and lifestyle. Meanwhile, although progression in anti-rheumatic therapies has been remarkable in the past two decades, favorably affecting quality of life for patients, these treatments such as conventional synthetic disease-modifying anti-rheumatic drugs(csDMARDs), biologic or targeted synthetic DMARDs (b/tsDMARDs) could also be carcinogenic. Here we summarized the risk of malignancies in systemic rheumatic diseases and the carcinogenic possibility of antirheumatic therapies, especially the newly developed b/tsDMARDs.

## 2 Systemic lupus erythematosus (SLE)

SLE is a systemic autoimmune disease, characterized by aberrant activity of the immune system, leading to varying clinical manifestations in the affected organs (2). SLE is proved to be linked with a slight increase in the overall risk of malignancy. According to previous reports, the standardized incidence ratio (SIR) for overall cancer risk in SLE population is 1.14 (95% CI 1.05-1.23) to 4.13 (95% CI 2.26-6.93) (3, 4). The risk of many malignancies, such as vulva, lung, thyroid, and hematologic cancers, particularly non-Hodgkin lymphoma (NHL), increased in varying degrees (4). However, prostate cancer, cutaneous melanoma, breast and endometrial cancer seem to be associated with a decreased risk in patients with SLE (5, 6).

Many potential risk factors have been related to the increase of certain malignancies in SLE patients. It is found in a large multi-center cohort of SLE patients that male sex and older age of SLE onset were risk factors for most cancer types (7). Another important risk factor for cancer, especially lung cancer in SLE population is smoking (7). Although cyclophosphamide was considered as a risk factor for nonmelanoma skin cancer (NMSC), other immunosuppressive medications for treating SLE were not clearly associated with higher risk of malignancies in these patients (7).

Both genetic predisposition to impaired DNA repair and chronic inflammation may facilitates the accumulation of DNA damage in patients with SLE, and thus partly contribute to the increased risk of certain malignancies (8). Besides, higher risk for virus-associated cancers (e.g., vaginal, cervical, anal cancers associated with HPV) in SLE suggests that inability to clear oncogenic infections may be one of the potential mechanisms linking cancer with this disease (9). As for the trend of decreased risk in other malignancies such as melanoma and breast cancer, particularly oestrogen-receptor-negative breast cancers, one possible explanation is that some DNA-damaging lupus autoantibodies are selectively toxic to cancer cells and tumors with pre-existing defects in DNA repair. Thus, these autoantibodies might contribute to a reduced incidence of DNA-repair-deficient malignancies in SLE (8).

## 3 Primary Sjogren’s syndrome(pSS)

Sjögren’s syndrome(SS) is a systemic autoimmune disease in which immune cells attack and destroy exocrine glands, resulting in classic symptoms of dry eye and mouth (10). It is divided into two categories: secondary SS which develops in addition to other autoimmune diseases and pSS which we focus here in this review. Numerous previous studies have confirmed that the risk of lymphoma in patients with pSS was increased (11, 12). It has been reported that these patients have a 5–15 times higher risk of lymphoma than the general population (13). Moreover, the risk of lymphoma development in pSS is the highest among all the autoimmune diseases (14).

Many predictive markers for the development of lymphoma in SS have been suggested. Hypocomplementemia and lymphocytopenia at pSS diagnosis are the strongest predictors (15). Parotidomegaly, purpura, splenomegaly, lymphadenopathy, anemia, rheumatoid factor(RF), cryoglobulinemia, presence of ectopic germinal centers, focus score of >3, score of >5 on the EULAR Sjögren’s Syndrome Disease Activity Index (ESSDAI), and germinal mutations in *TNFAIP3* have also been found as significant predictors for the development of lymphoma in patients with SS (10, 15).

Abnormal lymphoproliferation in SS is considered to be associated with lymphoma development. It is a multistep and chronic process during which a benign polyreactive B cell population is finally driven into a malignant monoclonal B cell component (16). Accumulated evidence have shown that chronic antigenic stimulation by exoantigen or autoantigens is of critical importance in this process (17).

Besides lymphoma, a recent meta-analysis showed that pSS was significantly associated with increased risks of overall malignancy(pooled SIR 2.17, 95% CI 1.57-3.00), hematological malignancy(pooled SIR 11.55, 95%CI 4.32-30.90) including NHL, Hodgkin lymphoma, multiple myeloma(pooled SIR 8.27, 95%CI 3.08-22.24), leukemia(pooled SIR 2.56, 95%CI 1.78-3.69) and solid tumors(pooled SIR 1.39, 95%CI 0.90-2.13) including lung cancer(pooled SIR 1.55, 95%CI 1.29-1.85), thyroid cancer(pooled SIR 2.05, 95%CI 1.20-3.48), NMSC (pooled SIR 1.71, 95%CI 1.08-2.72), kidney/urinary tract cancer(pooled SIR 1.36, 95%CI 1.02-1.81), liver cancer(pooled SIR 1.70, 95%CI 1.13-2.57) and prostate cancer(pooled SIR 1.50, 95%CI 1.02-2.22) (18). Data from the French health insurance database reported similar results, but found bladder and breast cancer incidences were lower in hospitalized pSS patients in France (19).

## 4 Idiopathic inflammatory myopathies (IIM)

IIM are a heterogeneous group of autoimmune disorders usually characterized by chronic inflammation of the muscle with variable clinical symptoms, treatment responses and prognosis (20). The subgroups of IIM include polymyositis (PM), dermatomyositis (DM), inclusion body myositis (IBM), antisynthetase syndrome (ASyS), and immune-mediated necrotizing myopathy (IMNM) (20). IIM has an increased risk of cancer, generally in the subgroups of PM and DM, especially the later (21). Approximately one in four IIM patients are diagnosed with cancer within 3 years before or after IIM onset (22).

The risk factors of malignancies in patients with IIM have been investigated in many studies. A recent meta-analysis showed that among IIM, DM subtype, older age, male sex, dysphagia, cutaneous ulceration and anti-transcriptional intermediary factor-1 gamma(anti-TIF1-γ) positivity were all associated with significantly increased risk of cancer, while PM and clinically amyopathic DM(ADM) subtypes, Raynaud’s phenomenon, interstitial lung disease, very high serum creatine kinase or lactate dehydrogenase levels, and anti-Jo1 or anti-EJ positivity were associated with reduced risk (23).

Although DM has been proved to be the subgroup most strongly associated with cancer in IMM, it is, actually, also comprised of a heterogeneous group with different cancer risk. ADM is a subtype within the DM spectrum which lacks the clinical as well as laboratory evidence of muscle involvement. The most commonly reported malignancies in ADM are breast, lung, and ovarian cancer, while nasopharyngeal cancer is among the three most common ones in “classic” DM besides lung and ovarian cancer (24). Moreover, the discovery of myositis specific antibodies(MSA) has revealed that those subtypes can be distinguished from one another based on serology (25). Numerous studies have suggested the presence of anti-TIF1-γ confers a great risk of cancer development in DM (26–28).And data from a large UK-based adult DM cohort found that cancer types differ according to anti-TIF1-Ab status, with female anti-TIF1-Ab-positive patients at increased risk of ovarian cancer (26). Among anti-TIF1-γ-positive DM patients, coexisting autoantibodies against transcription factor Sp4 and cell division cycle and apoptosis regulator protein 1 (CCAR1) are associated with decreased cancer risk (29, 30).

The mechanisms linking IIM to the onset of tumor have not yet been fully understood, yet studies have proposed that cancer-DM relationship is a continuum that can be explained within the framework of cancer immunoediting and a cancer triggered autoimmune mechanism is involved in the pathogenesis of IMM, especially DM (30, 31). One possible mechanism is that antitumor response targeting self-proteins mutated in tumors could be directed toward skeletal muscle cells. Another possible mechanism is that autoantigen overexpression by tumor cells could initiate the process of autoimmunity targeting muscle and other related tissues (31, 32). A recent study demonstrated an increased number of genetic alterations in TIF1 genes of tumors from paraneoplastic anti-TIF1-γ-positive patients, as well as a high expression of TIF1g in the tumor, muscle and skin of these patients. This study suggested that these two facts would be important to understand the genesis of paraneoplastic myositis (33).

## 5 Systemic sclerosis (SSc)

SSc is a relative rare rheumatic disease with vascular injury and fibrosis as its main clinical features (34). According to a study from the European Alliance of Associations for Rheumatology (EULAR) Scleroderma Trials and Research (EUSTAR) database, malignancy has become one of the leading causes of death in this disease (13%) (35). An Scleroderma Cohort Study showed that SSc patients have more than two fold higher risk of cancer occurrence than age- and sex- matched population peers in Australian (36). And the most common cancers in SSc patients are lung cancer and hematological malignancies (37, 38).

A study by Igusa et al. suggested that cancer risk was different in different subgroups according to SSc phenotype, autoantibodies, and temporal clustering (39). In this study, seronegative patients and anti-RNA-Pol-III-positive patients were found to have a higher risk for malignancy, while anticentromere-positive patients had a lower risk. Another research showed that PM/Scl antibodies seem to be associated with a higher risk of cancer in SSc (40).

The association between SSc and malignancy is complex. On one side, chronic inflammation, as well as several environmental factors and occupational exposures such as crystalline silica, organic solvents, pollutants, pesticides, etc., have been regarded inducers of tumor in SSc patients (41, 42). On the other side, it has been suggested that the “foreign” antigen triggering the autoimmune response in SSc patients might actually be a tumor antigen (43).

## 6 Rheumatoid arthritis (RA)

RA is a chronic autoimmune disease characterized by inflammation and destruction of cartilage and bone in affected joints (44). The association between RA and malignancy was first reported in 1978 (45). Since then, many studies have investigated the association. A meta-analysis by Simon and colleagues confirmed that compared with general population, patients with RA has a modest increased risk (10%) in overall malignancy (46). Also, site-specific malignancy risks were reported. The risk of lung cancer as well as lymphoma was greatly increased. In contrast, the risk of colorectal cancer and breast cancer was decreased (46).

One theory which might partly explain the increased incidence of certain cancer in RA patients is that both these cancers and RA share similar risk factors (47). For example, smoking is a risk factor for both RA and lung cancer (48, 49). It is also suggested that chronic immune stimulation/chronic inflammation and damage in RA may lead to the increased risk of cancer, which supported by the facts that elevated inflammatory biomarkers such as ESR and CRP were associated with increased cancer risk; whereas longer duration corticosteroid therapy were found to be linked with lower lymphoma risk (9, 50). 

With the increasing use of immunosuppressive DMARDs and newly developed biologic agents in the management of RA, the clinical outcomes have been greatly improved (51). However, whether or not the treatment for RA can increase the risk of cancers has been of great interest (47).

## 7 Anti-rheumatic therapies

### 7.1 Nonsteroidal anti-inflammatory drugs (NSAIDs) and glucocorticoids(GCs)

Many studies have demonstrated that inflammation can predispose to tumors, thus people have been interested in whether targeting inflammation and the molecules involved in the inflammatory process could represent a good strategy for cancer prevention and treatment. NSAIDs, as well as some other anti-inflammatory agents, have been demonstrated to be able to reducing cell migration and increasing apoptosis and chemo-sensitivity in the tumor microenvironment in many clinical studies (52). Moreover, several researches also suggested that NSAIDs have the potential to act as chemoprevention therapy or anticancer agents in certain malignancies (52–54).

Treatment with glucocorticoid does not appear to be associated with an increased incidence of some malignancies like lymphomas (55–57). However, studies have demonstrated that GCs have complicated influence on pathophysiological processes related to malignancy which needs further investigation (58).

### 7.2 csDMARDs

Among csDMARDs, cyclophosphamide(CTX) is the most well-known carcinogenic agent which is associated with the risk of bladder cancer, as well as secondary acute leukemia and skin cancer (59).

Methotrexate (MTX) entered clinical medicine as an innovative anti-neoplastic drug in 1948, and later it became favored over cyclophosphamide by rheumatologist in treating progressive RA because of its clinical benefit and lack of carcinogenicity (60). There is no evidence to confirm any oncogenicity effects of MTX, although some case reports have linked it to lymphomas and pseudo lymphomas (61). Of note, Solomon and colleagues observed in their study that non-biologic DMARDs other than MTX (such as leflunomide, sulfasalazine, or hydroxychloroquine) were associated with a reduced overall cancer risk compared with MTX (Hazard Ratio 0.17, 95% CI 0.05-0.65), as well as for several specific cancers (62).

Mycophenolate is an immunosuppressive drug approved for the prophylaxis of allograft rejection in transplant recipients. It is now widely used in many autoimmune disorders. Generally, it does not show a discernable cancer trend (63), and may even have an antitumor effect for several human cancers, including leukemia and some solid tumors (64).

Azathioprine is also a frequently used csDMARDs. Azathioprine treatment seems to be safe with regards to the risk of malignancy in the SLE population (65), but is likely to be linked with lymphoma development in RA patients (66). A 20 year follow-up study showed that although there was a fivefold increase in the lymphoma rates in azathioprine treated RA patients compared with the general population, this increased risk was still small in relation to the relative increase in “background” risk in the RA population (66).

Low dose of cyclosporine has been used in the treatment of autoimmune diseases, including RA and SLE. Several studies have evaluated its oncogenic possibility in nontransplant patients and the results are conflicting (67, 68). However, the majority of them did not find it related with a higher risk of malignancy, especially compared with other csDMARDs (67–69).

### 7.3 bDMARDs

Many studies have showed that there was no increased risk of malignancies in RA patients with bDMARDs compared with the general population, except for melanoma and NMSC (70–72).And the risk of most malignancies was not increased in patients treated with bDMARDs compared with those on csDMARDs (73). However, the association between certain bDMARD with urinary tract cancer still needs further investigation, as a recent report from the anti-Rheumatic Treatment in Sweden Register(ARTIS) group reported a statistically significantly increased risk of urinary tract cancer with Tumor necrosis factor inhibitors (TNFi), rituximab(RTX) as well as abatacept(ABA) when comparing site-specific relative risks for cancer with b/tsDMARDs vs. b/tsDMARD naïve RA (74).

#### 7.3.1 Tumor necrosis factor inhibitors (TNFi)

TNFi (such as adalimumab, certolizumab pegol, etanercept, golimumab, and infliximab) are widely used to treat chronic inflammatory conditions, including RA, juvenile idiopathic arthritis, psoriasis, psoriatic arthritis, spondylarthropathies, inflammatory bowel disease and uveitis (75). However, historically, whether anti-TNFα therapies predispose people to cancer has been deeply concerned (76, 77), as the relationship of tumor necrosis factor (TNF), a multifunctional cytokine, with tumor is complex. On one hand, TNF has undisputed tumor-destructive function under certain circumstances; on the other hand, it is a major mediator of cancer-related inflammation and may exert tumor-promoting effects (77). As a result, TNFi was often avoided in patients with a history of malignancy.

In a large observational study by ARTIS group, Wadström and colleague found that the overall cancer risk among RA patients initiating treatment with TNFi was similar compared with that among patients with bDMARD-naïve RA (70). Moreover, according to a nationwide, population-based cohort study, treatment with TNFi was not associated with recurrent or new primary cancer development in patients with previous cancer (78). With respect to site-specific malignant neoplasms, the risk of skin cancer, particularly NMSC, in patients who recieved TNFi is of greatest concern and is still controversial (79–81).

#### 7.3.2 Rituximab(RTX)

RTX is a CD20 specific murine/human chimeric monoclonal antibody agents. Since registered by the Food and Drug Administration in 1997 for treatment of lymphomas, it has improved the prognosis of all B-cell derived lymphoproliferative diseases (82). It has also been used in the treatment of many rheumatic diseases, such as ANCA-associated vasculitis (83). A systematic literature review showed that RTX did not increase the risk of cancer in patients who received this therapy (73).

#### 7.3.3 Tocilizumab(TCZ)

TCZ, an IL-6 receptor antagonist, has been approved for the treatment of RA in many countries throughout the world (84).Compared with csDMARDs, TCZ does not increase the risk of overall cancer, as well as solid cancer (excluding NMSC), NMSC, melanoma, and haematological cancer (70).

#### 7.3.4 Abatacept(ABA)

CTLA-4 agonist ABA is a fusion protein approved for the treatment of RA. It binds to CD80 and CD86 receptors on antigen presenting cells, and then inhibits T-cell proliferation and B-cell stimulation (85). A world observational post-marketing study investigated the risk of overall malignancy and specific cancers in RA patients who received ABA. It was found that compared with other bDMARDs, exposure to ABA was only significantly associated with an increased risk of melanoma. It is not surprising since ABA has an opposite action than ipilimumab, an approved drug which treats malignant melanoma by blocking CTLA-4 (86).

### 7.4 tsDMARDs

tsDMARDs are made from synthetic chemical compounds. However, unlike csDMARDs which have a dampening effect on the whole immune system, tsDMARDs target specific parts of the immune system, like bDMARDs. In RA and many other rheumatic diseases, the only approved tsDMARDs are Janus kinase (JAK) inhibitors (87). Currently licensed JAK inhibitors include tofacitinib (Pfizer, New York, NY, USA), ruxolitinib (Novartis, Basel,Switzerland), fedratinib (Celgene, Summit, NJ, USA), upadacitinib (AbbVie, North Chicago, IL, USA), peficitinib (Astellas Pharma, Tokyo, Japan), and baricitinib (Eli Lilly, Indianapolis, IN, USA) (88). Among them, tofacitinib, baricitinib, upadacitinib and fedratinib have been approved for treating rheumatic diseases, and peficitinib has only been approved for RA treatment in some Asian countries.

Tofacitinib is a type of JAK inhibitor that preferentially inhibits JAK1 and JAK3. Whether or not tofacitinib increases the risk of developing cancer has been controversial, and the results of studies may be affected by factors such as follow-up time, the definition of the study population, exposure and outcome definition. Maneiro and colleagues showed in a meta-analysis that in RCTs, tofacitinib does not increase the risk for malignancy in RA patients (89). Another meta-analysis also confirmed that there was no increased risk of developing cancer in general or specific cancer types in RA patients receiving treatment with tofacitinib, compared with those receiving csDMARDs or TNFi (90). Recently, however, an open-label, randomised controlled trial (ORAL Surveillance; NCT02092467) which assessed the safety of tofacitinib in comparison with TNFi in patients 50 years of age and older, with at least one cardiovascular risk factor, and with background MTX treatment showed that incidence rates (IRs) for malignancies excluding NMSC and NMSC were higher with tofacitinib versus TNFi (91). Besides, Khosrow-Khavar and colleagues also reported that compared with TNFi, tofacitinib was associated with a numerically increased risk of malignancies(pooled weighted HR: 1.17, 95% CI: 0.85, 1.62) in a subgroup of “RCT-duplicate” RA cohort which emulated the inclusion and exclusion criteria of the ORAL surveillance trial, although it was not associated with the risk in the whole population-based real-world cohort of 83,295 RA patients (92).

Baricitinib is a reversible and selective JAK1/JAK2 inhibitor for treating RA. According to the result from an integrated database of patients who received any baricitinib dose (All-bari-RA), the IR for malignancy (excluding NMSC) during the first 48 weeks was 0.6 (95% CI 0.34-0.91) and remained approximately 1.0 (overall IR 0.9, 95% CI 0.77-1.09) thereafter. The overall age-adjusted SIR was 1.07 (95% CI 0.90-1.26), suggesting comparable incidence of malignancies with the general US population (93).

Upadacitinib is a JAKi engineered for greater JAK1 selectivity over other JAK family members. Integrated analysis from the SELECT phase III clinical programme revealed that rates of malignancies in RA patients receiving upadacitinib were similar among those receiving upadacitinib, MTX or adalimumab (94).

## 8 Mechanisms linking malignancies and rheumatic diseases

**Figure 1:**
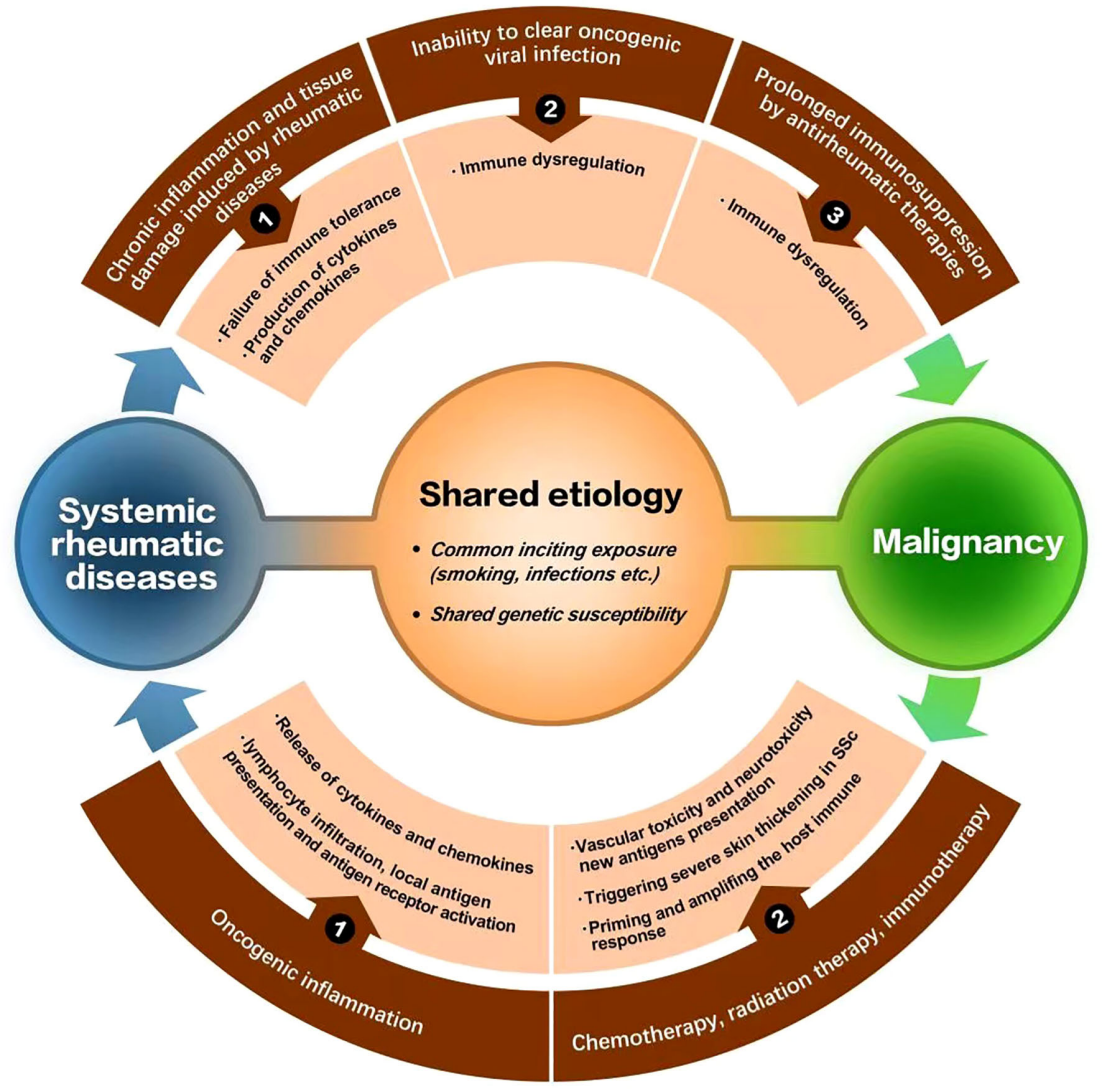
Mechanisms linking malignancies and rheumatic diseases.

Although possible mechanisms for tumor incidence in systemic rheumatic diseases have been discussed in each section above, several potential mechanisms linking malignancies and rheumatic disease have been generally suggested up to now (9, 95, 96). Chronic inflammation and tissue damage induced by rheumatic disease, inability to clear viral oncogenic infections and certain therapies discussed above are related to malignancies secondary to rheumatic disease; while cancer-induced autoimmunity, treatments for cancer (immunotherapy, chemotherapy or radiation therapy) may contribute to the occurrence of rheumatic disease secondary to malignancies. Besides, shared etiology such as common inciting exposure or shared genetic susceptibility is also a potential mechanism (**Figure 1**) (9, 95, 96).

## 9 Discussion

Questions relating to malignancy and rheumatic disease arise commonly in clinical practice. The occurrence of tumor in patients with systemic rheumatic diseases is not rare and negatively impact on their quality of life. The risk and types of malignancies differ in different systemic rheumatic diseases, and even in different subgroups of one particular rheumatic disease. Predictive markers for the development of malignancy in different rheumatic diseases and possible mechanisms linking them have been suggested, which still need further investigation in the coming years.

Generally, the majority of DMARDs was not associated with increased risk of most malignancies in patients with systemic rheumatic diseases. Since most of these patients need to receive DMARDs, and some even need b/tsDMARDs in order to attain and maintain adequate control of the disease, our work may help clinical decisions when regarding malignancy risk of these therapies for them. However, it is of note that some of the above results come from RCTs, especially for newly developed therapies such as JAK inhibitors, and RCTs are not appropriate for assessing the malignancy risk of long-term drug exposure. Therefore, further studies with long-term follow-up and large sample size are still needed for these drugs. And in clinical practice, the risk-benefit ratio should always be fully considered when selecting therapeutic regimen for these patients, so as to improve the long-term prognosis.

## Author contributions

Writing - original draft, ZG and CY; Writing - review & editing, and funding acquisition, XZ. All authors contributed to the article and approved the submitted version.
